# A Prediction Model for Instability in Adult Distal Radius Fractures: Integrating Post-Reduction and Follow-Up Indicators

**DOI:** 10.3390/jcm14238336

**Published:** 2025-11-24

**Authors:** Nuttapol Khajonvittayakul, Kittiwan Supichyangur, Adinun Apivatgaroon, Pichaya Tantiyavarong

**Affiliations:** 1 Department of Clinical Epidemiology, Faculty of Medicine, Thammasat University, Pathum Thani 12120, Thailand; nuttapolcoach@gmail.com; 2 Department of Orthopaedic, Tharongchang Hospital, Surat Thani 84130, Thailand; 3 Hand and Reconstructive Microsurgery Unit, Department of Orthopaedic Surgery, Rajavithi Hospital, College of Medicine, Rangsit University, Bangkok 10400, Thailand; kittiwan.s@rsu.ac.th; 4 Department of Orthopaedic, Faculty of Medicine, Thammasat University, Pathum Thani 12120, Thailand; adino_ball@yahoo.com

**Keywords:** distal radius fractures, fracture instability, prediction model, Lafontaine criteria

## Abstract

**Background/Objectives**: Although Lafontaine criteria are widely used to predict fracture instability for distal radius fractures (DRFs), their predictive performance remains limited. This study aimed to enhance prediction accuracy by incorporating post-reduction and one-week follow-up radiographic findings. **Methods**: This retrospective study included adults with DRFs treated with closed reduction and casting. A predictive model was developed using pre- and post-reduction radiographs through stepwise multivariable logistic regression. Simplified scores were derived to classify patients into low-, moderate- and high-risk groups, guiding follow-up or early surgical intervention. An additional predictive model based on one-week radiographs was developed for the moderate-risk group with uncertain stability. Internal validation was performed using bootstrapping, and model performance was compared with Lafontaine criteria. **Results**: Of 402 patients identified, 244 met inclusion criteria; 161 developed malalignment and 98 required surgery. The mean age was 58.5 ± 16.7 years, and 75.1% were female. In baseline model, significant predictors of instability included dorsal angulation > 20°, intra-articular fracture, ulnar variance > 3 mm, volar cortex restoration, and post-reduction volar angulation ≤ 0°. Internal validation demonstrated good performance (optimism-adjusted AUC = 0.86). Risk stratification identified 39% of patients as moderate risk, who were subsequently used to develop a one-week follow-up model, with ulnar variance > 3 mm as a key predictor for instability. The overall model outperformed Lafontaine criteria (AUC = 0.75 vs. 0.68). **Conclusions**: The proposed model effectively stratifies instability risk and supports clinical decision-making by integrating critical post-reduction and one-week radiographic parameters, offering greater accuracy than existing criteria.

## 1. Introduction

Distal radius fractures (DRFs) are among the most common orthopedic injuries [1], accounting for approximately 20% of all fractures treated in emergency departments [2,3]. Although closed reduction and casting remain standard treatments [4,5], post-reduction displacement occurs in 43–64% of cases [6,7,8], frequently requiring surgical intervention. Early identification of patients at high risk of displacement is essential because delayed surgical intervention may lead to poorer functional outcomes, increased wrist stiffness, skin complications, and more technically challenging procedures. Nevertheless, accurately identifying these high-risk patients remains challenging [7].

Several criteria have been proposed to predict DRF instability, most notably the Lafontaine criteria [9], which suggests instability when multiple factors—such as dorsal angulation, dorsal comminution, or advanced age—are present. Although frequently referenced in clinical decision-making, their predictive validity has been increasingly questioned, with multiple studies reporting inconsistent or limited accuracy [3,6,8,10,11]. Other models, including those by MacKenney et al. and Adolphson et al. [11], have also demonstrated restricted clinical utility.

In clinical practice, follow-up radiographs are obtained after closed reduction to assess reduction adequacy and monitor for early redisplacement, typically at the one-week visit. Evidence shows that factors beyond initial fracture characteristics—such as reduction quality [12], bone quality [13], and early displacement during follow-up [14,15]—play critical roles in predicting instability. Despite these insights, most existing predictive models rely mainly on pre-reduction radiographic parameters and overlook these dynamic post-reduction predictors. This study aimed to develop and internally validate a predictive model and scoring system for fracture instability after closed reduction by integrating a broader range of clinical and radiologic factors to improve early risk stratification and support treatment decision-making.

## 2. Materials and Methods

### 2.1. Data and Participants

This retrospective cohort study was conducted from 1 January 2014 to 31 December 2022 and included adults with DRFs treated with closed reduction and immobilization. Data were obtained from two referral hospitals, both of which have hand orthopedic specialists: Rajavithi Hospital, a 1200-bed tertiary care center, and Tharongchang Hospital, a 90-bed general hospital. The study was approved by the Human Research Ethics Committee of Rajavithi Hospital (COA No. 185/2023) and the Ethics Committee of Thammasat University (Project No. MTU-EC-ES-4-162/66). It was conducted in accordance with the TRIPOD reporting guidelines.

Eligible participants were identified from hospital databases using ICD-10 codes and included adults (≥18 years) with DRFs treated non-operatively. Diagnosis was confirmed clinically and by standard wrist radiographs (posteroanterior and lateral views) performed on the day of injury. Exclusion criteria were skeletal immaturity, existing indications for immediate operative treatments (such as open fractures, volar Barton fractures, severe intra-articular fractures, or multiple trauma), inadequate follow-up before fracture union, incomplete medical records, and cases in which surgery was performed due to patient or surgeon preference rather than established operative indications (e.g., desire for quicker recovery, concern about deformity, denial of casting, or surgeon preference despite acceptable alignment). However, patients who declined a recommended operation were retained in the analysis and classified as having achieved the outcome of fracture instability. These exclusions were applied to ensure a homogeneous cohort for prediction modeling and to minimize confounding due to variations in treatment.

All patients received standardized non-operative management, including hematoma block anesthesia, closed anatomical reduction by longitudinal traction, and immobilization with either a U-shaped or anterior–posterior short arm splint. The wrist was positioned in neutral to slight extension with ulnar deviation. Splints were applied by orthopedic surgeons or residents in training under supervision. They were converted to below-elbow casts once swelling subsided, typically within 1–2 weeks. Splint molding was performed followed by the three-point fixation principle to maintain anatomical alignment. All included patients had initial, post-reduction, and one-week follow-up radiographs. Subsequent imaging was performed at the discretion of the attending physician based on the degree of alignment and perceived stability, with follow-up typical occurring at two, four and six weeks until either failure or successful completion of conservative treatment was established. For patients who developed unacceptable alignment, casts were removed once the decision for surgical intervention was made. In contrast, among those who remained in nonoperative management, casts were removed no later than four weeks since signs of stabilization appeared—defined as trabecular bridging across the fracture site—preceding complete union. A final radiograph was obtained at the six-week follow-up visits.

### 2.2. Outcomes and Predictors Measurement

The primary outcome was fracture instability, defined as the presence of unacceptable alignment on the final radiographic assessment—either at the six-week follow-up visit or on the immediate pre-operative radiograph for patients who underwent surgery after unacceptable alignment was identified at the two- or four-week visit. Unacceptable alignment was defined as the presence of at least one of the following criteria: radial inclination (RI) < 10°, radial shortening (RS) > 4 mm, volar angulation > 20°, dorsal angulation (DA) > 10°, or intra-articular fracture stepping (IAFS) > 2 mm.

Potential predictors of instability were categorized by time point. Initial factors included Lafontaine criteria (age > 60 years, ulnar fracture, DA > 20°, IAFS, dorsal comminution), as well as other variables: ulnar variance (UV) > 3 mm, second metacarpal cortical percentage (2MCP) ≤ 50%, RI ≤ 10°, radial height ≤ 11 mm, metaphyseal comminution, and distal radioulnar joint (DRUJ) separation. Post-reduction factors included volar cortex restoration, residual volar angulation, DRUJ separation, radial translation, and splint quality (quantified by three-point molding distance on lateral radiographs). One-week follow-up factors included re-assessment of volar angulation and UV.

**Figure 1:**
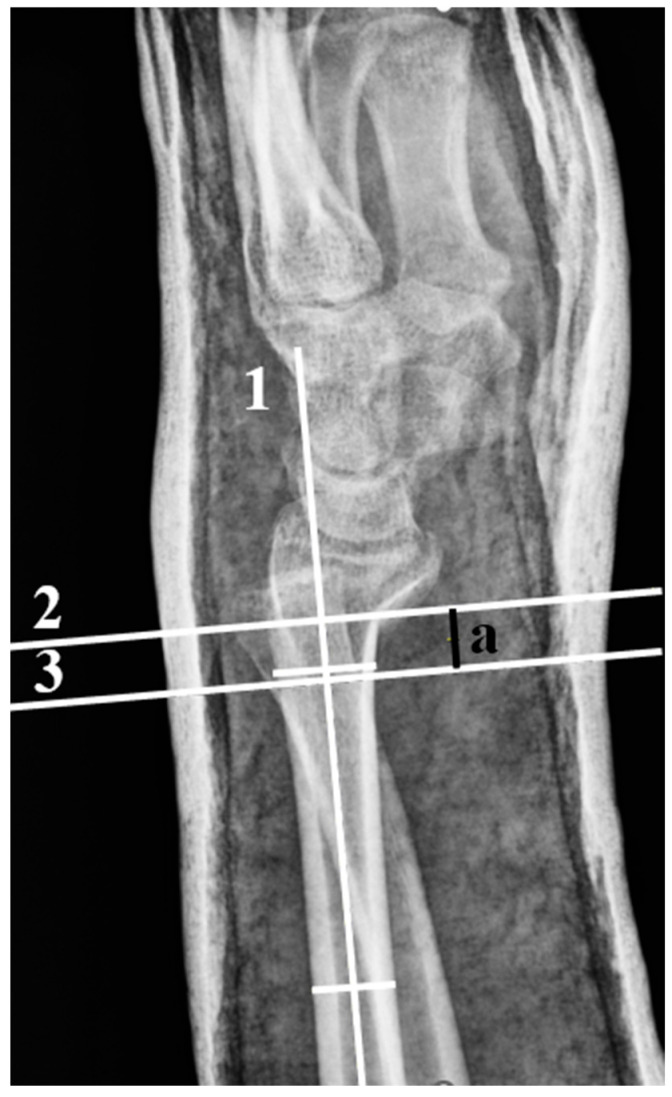
Measurement of Three-Point Molding Distance (a). The long axis of the radius is identified (1). A perpendicular line to this axis is drawn at the fracture site on the volar cortex (2) and at the apex, or narrowest point, of the volar splint (3). The distance is considered positive when the splint’s narrowest point lies proximal to the fracture site.

Radiographic assessments were based on standardized posteroanterior and lateral views, as shown in Tables S1 and S2. Figure 1 shows the three-point molding distance measured after reduction. All radiographic measurements were performed on calibrated PACS software (v.5.7.100 at Rajavithi Hospital and v.3.0.11.5_B5P0 for Tharongchang Hospital), with angular accuracy within 1° and linear within 1 mm. Two independent hand and microsurgery specialists (N.K., 2 years’ experience; K.S., 13 years’ experience) conducted all measurements following a standardized protocol.

Interobserver reliability was assessed on a random sample of 20 cases, with raters blinded to each other’s evaluations, and showed good to excellent agreement. Data were entered into REDCap electronic forms [16,17] and analyzed using de-identified datasets to minimize selection and information bias.

### 2.3. Sample Size

The sample size was calculated using the rule of 10 outcome events per predictor variable [18]. Previous reports have shown that the instability rate is approximately 47–64% [6,7]. Assuming an anticipated instability rate of 60% and 14 candidate predictors, a minimum of 234 participants was required [6,7].

### 2.4. Statistical Analysis

Because all radiographic images were measured following a standardized protocol, no missing predictor data were present in the dataset. Analyses were conducted using STATA version 17.0, with significance set at *p* < 0.05 (two-sided). Categorical variables were summarized as frequencies and percentages, and continuous variables as means with standard deviations or medians with interquartile ranges, depending on distribution. Between-group comparisons used Chi-square or Fisher’s exact tests for categorical variables and independent *t*-tests or Mann–Whitney U tests for continuous variables.

Univariable logistic regression was used to assess the associations between each predictor and fracture instability. The linearity assumption between continuous predictors and the outcome was evaluated; DA, UV, and post-reduction DA demonstrated non-linear associations with instability and were therefore categorized prior inclusion. Collinearity among predictors was assessed using variance inflation factors. A multivariable logistic regression model was developed using initial and post-reduction factors, with stepwise backward elimination to retain predictors with *p* values < 0.05. Adjusted odds ratios (ORs) with 95% confidence intervals (CIs) were estimated for predictors retained in the final model.

Model discrimination was evaluated with the area under the receiver operating characteristic curve (AUC). Calibration was assessed using the Hosmer–Lemeshow goodness-of-fit test and calibration plots. Internal validation was performed with bootstrap resampling (500 iterations) to obtain optimism-adjusted AUC and calibration slopes.

The final model was converted into a clinical prediction score by assigning weights to each predictor. Weights were derived by standardizing the regression coefficients—dividing each coefficient by the smallest coefficient in the model—and then rounding to the nearest 0.5 or integer value to facilitate practical clinical use. The scores were tested for performance using AUC to ensure consistency with the original model and were categorized to stratify patients into low-, moderate-, and high-probability groups of instability, ensuring that both false positive and false negative rates remained below 5%. This clinical prediction rule was designed to guide patient management decisions based on risk stratification. Patients in the low-probability group were managed conservatively with closed reduction and splinting, while those in the high-probability group were advised to undergo early surgical intervention. Patients in the moderate-probability group, with uncertain instability, required close monitoring and reassessment at the one-week follow-up. A separate multivariable logistic regression model, incorporating two predictors available at the one-week follow-up, was developed. Finally, the performance of overall model was compared with Lafontaine criteria using AUC.

## 3. Results

### 3.1. Participants

Of 402 identified patients, 244 met the inclusion criteria and were included in the study. Among these, 161 (65.9%) demonstrated unacceptable alignment, of whom 98 underwent surgery (Figure S2). The mean age was 58.5 ± 16.7 years, and 75.1% were female. Baseline characteristics and radiographic parameters, including at baseline and one week, are summarized in Table 1.

### 3.2. Baseline Model

Univariable logistic regression analysis identified several factors significantly associated with instability (Table 2), including DA > 20°, IAFS, dorsal comminution, UV > 3 mm, restoration of the volar cortex, including dorsal and volar overlapping, volar angulation ≤ 0°; and DRUJ separation.

In the multivariable logistic regression, five independent predictors were retained: DA > 20° (OR, 3.74), IAFS (OR, 2.27), UV > 3 mm (OR, 5.97), restoration of the volar cortex —dorsal overlapping (OR, 2.34), and volar overlapping (OR, 4.35), and volar angulation ≤ 0° (OR, 4.78) (Table 3).

**Figure 2:**
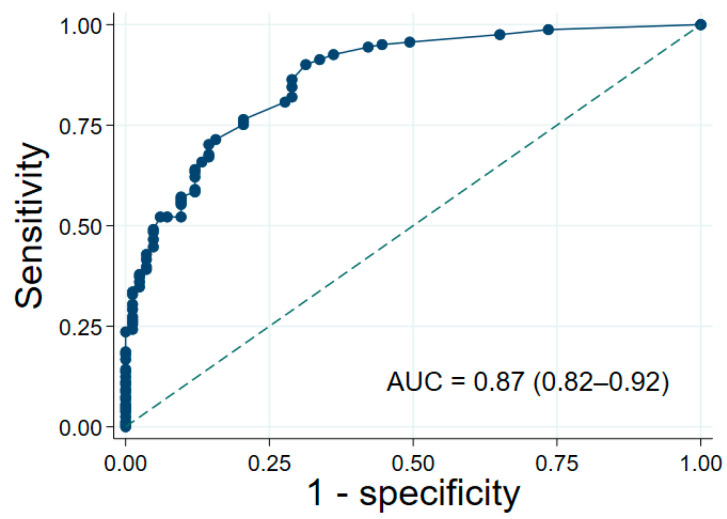
The Area Under the Curve (AUC) of final model. The diagonal dashed line indicates performance equivalent to random chance.

The model exhibited excellent discrimination, with an AUC of 0.87 (95% CI: 0.82–0.92; Figure 2) in the development cohort. A discrimination plot (Figure S2) clearly distinguished stable from instable groups. Calibration was supported by a non-significant Hosmer–Lemeshow test (*p* = 0.80), while a calibration plot (Figure S3) confirmed strong agreement between predicted probabilities and observed risks.

Internal validation using 500 bootstrap replications confirmed high model performance, with an optimism-adjusted AUC of 0.86 (95% CI: 0.81–0.90) and an optimism-adjusted calibration slope of 0.91 (95% CI: 0.64–1.18), indicating minimal overfitting.

### 3.3. Clinical Prediction Score and Risk Stratification

Predictive scores were generated by assigning weights to each predictor. Table 3 presents the final scoring system, with predictors grouped into initial factors and quality of reduction factors, including their coefficients, *p* values, and assigned scores. The AUC of the predictive scores was 0.87 (95% CI: 0.82–0.91), comparable to that of the original final model. Scores were analyzed across varying cutoffs, with sensitivity, specificity, and accuracy reported in Table 4. The scores were subsequently stratified into three clinical risk groups to guide clinical management: low probability for instability (≤ 2 points), moderate probability for instability (2.5–5 points), and high probability for instability (≥ 5.5 points).

### 3.4. One-Week Follow-Up Model

**Figure 3:**
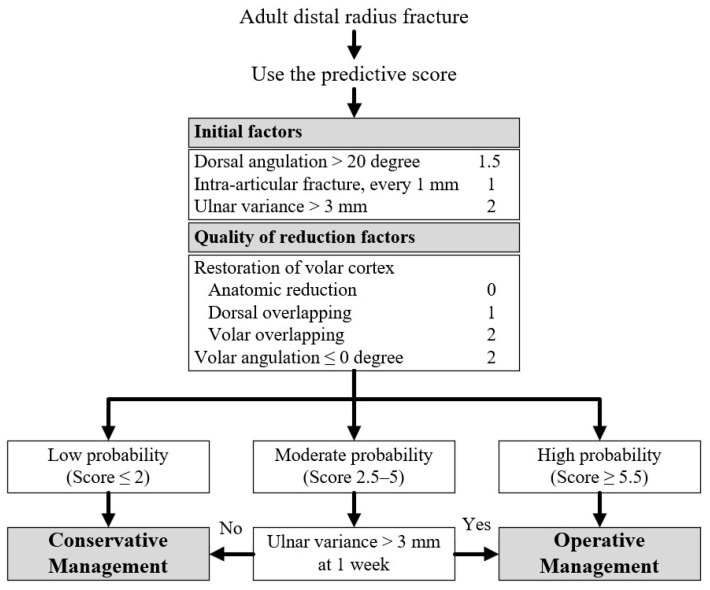
Clinical Decision Algorithm for Instability of Adult Distal Radius Fractures. The arrows illustrate the clinical decision pathway derived from the predictive scoring system.

Among 96 patients in the moderate probability group, multivariable logistic regression at one-week follow-up identified UV > 3 mm as the only independent predictor at one-week reassessment (OR, 9.67; *p* = 0.03; Table S3). Its diagnostic performance included LR+ 7.0, sensitivity 33.3%, specificity 95.2%, PPV 96.2%, and NPV 28.6%. Patients with UV > 3 mm at one week are recommended for surgical intervention; otherwise, conservative treatment is appropriate. The full clinical decision algorithm is summarized in Figure 3.

### 3.5. Comparison with Lafontaine Criteria

The newly developed model outperformed the Lafontaine criteria, achieving an AUC of 0.75 versus 0.68 (Figure S4). Lafontaine model, based on DA > 20°, dorsal comminution, intra-articular fracture, ulnar fracture, and age > 60, indicates instability if three or more criteria are present. These findings support the improved discriminative power of the new model in identifying unstable distal radius fractures, with potential for enhanced clinical decision-making.

## 4. Discussion

In this study, we developed and validated a predictive model for fracture instability that demonstrated strong discriminative ability and outperformed Lafontaine criteria. Key fracture characteristics, including DA, IAFS, inadequate volar cortex restoration, post-reduction volar angulation, and UV, were identified as significant predictors of instability. Risk stratification further highlighted a subset of patients at moderate risk who benefited from close follow-up, with early changes in UV at one week serving as a particularly important indicator of potential deterioration. These findings emphasize the value of incorporating initial, early post-reduction, and one-week radiographic parameters to guide clinical decision-making and optimize patient management.

The findings of this study reinforce that the Lafontaine criteria have limited accuracy in predicting distal radius fracture instability. Prior research has increasingly questioned their predictive validity. Nesbitt et al. [3] observed that intra-articular fracture and dorsal comminution did not contribute to instability as originally proposed. Alemdaroǧlu et al. [10] found no significant association between any of the Lafontaine factors and instability. More recent validation studies demonstrated limited clinical utility and low predictive accuracy [6,8,11]. Our results further support and refine this body of evidence by showing that, although Lafontaine et al. [9] originally identified five predictive factors, only a subset appears to maintain consistent relevance in contemporary practice. In particular, DA > 20° and IAFS emerged as the most reliable predictors in our analysis, aligning with findings from several prior investigations [15,19,20]. This convergence underscores that pronounced DA and intra-articular displacement are robust indicators of biomechanical instability across diverse patient populations and clinical contexts.

In contrast, variables such as patient age, ulnar fracture, and dorsal comminution—long debated in the literature—did not demonstrate independent predictive value in our cohort. While age is commonly incorporated into prior risk assessment tools, our findings, consistent with several recent studies [3,5,7,21,22], indicate that chronological age alone does not adequately reflect the mechanical factors driving fracture instability. Likewise, the inconsistent associations reported for ulnar fracture and dorsal comminution in earlier cohorts, together with our results, further challenge the notion that these features possess independent prognostic significance [7,10,22].

Baseline UV > 3 mm emerged as one of the strongest predictors of fracture redisplacement, consistent with findings from Mackenney et al. [8] and Ghodasra et al. [13]. In addition to this intrinsic fracture characteristic, our results highlight role of reduction quality in determining stability. Specifically, lack of volar cortex restoration and post-reduction volar angulation ≤ 0° were both significant predictors of early loss of alignment. The importance of restoring the volar cortex is well supported by prior studies [12,15,23]. Moreover, recent evidence further emphasizes the prognostic value of achieving acceptable dorsal angulation following reduction [13,14,24]. Collectively, these findings support a model in which inherent fracture morphology establishes baseline instability risk, while modifiable technical aspects of the reduction further influence the likelihood of redisplacement.

Splints were used in this study as our standard method of distal radius fracture immobilization, offering stabilization and swelling accommodation. Although cast-based indices have been reported to be associated with fracture instability, our evaluation using the three-point molding distance showed no predictive value [10]. This may be explained by the consistent adequacy of splint positioning in both groups, with post-reduction alignment factors exerting a greater influence.

When compared with existing tools, our newly developed scoring system demonstrated superior discriminative ability relative to the Lafontaine criteria. This improvement likely reflects several factors: (i) prioritizing the most informative Lafontaine features (DA > 20°, IAFS), (ii) incorporation of UV > 3 mm as a key baseline predictor absent from the original criteria, and (iii) and explicit consideration of post-reduction quality indicators. However, requiring radiographic measurements at both pre- and post-reduction may make the model slightly less convenient for routine clinical use. Although the observed AUC for Lafontaine criteria in our cohort was higher than in some prior validations [25]—potentially due to differences in patient populations or outcome definitions—the consistent outperformance of the new model across analyses underscores its enhanced clinical utility.

A key strength of this study is the exclusive reliance on standard radiographs routinely obtained in clinical practice, which avoids additional radiation exposure and ensures the model’s direct applicability to real-world workflows. Importantly, our findings refine the set of reliable predictors by incorporating both baseline fracture morphology (DA > 20°, IAFS, UV > 3 mm) and post-reduction quality indicators (volar cortex restoration, post-reduction volar angulation ≤ 0°), along with one-week UV > 3 mm, into a clinically practical prediction rule.

Nonetheless, several limitations should be acknowledged. The retrospective design may introduce selection bias, and exclusion of patients with incomplete radiographs or loss to follow-up could limit the external generalizability of our findings. Additionally, because the study was conducted at referral centers, the proportion of unstable fractures may have been higher than in general practice, potentially influencing the observed prevalence and predictive performance. Furthermore, inconsistent documentation of injury dates required the use of initial radiograph dates as a proxy, which may have introduced minor misclassification. Importantly, variables such as time from fracture to treatment and comorbidities, including diabetes, were not systematically collected and therefore could not be evaluated or incorporated into the predictive model.

## 5. Conclusions

This study presents a validated and pragmatic prediction model for assessing instability in initially well-aligned DRFs. The model demonstrates superior predictive accuracy compared with prior criteria and highlights modifiable factors where clinicians can intervene to optimize outcomes. Future research should prioritize external validation in broader, community-based populations and prospective studies to confirm generalizability, evaluate performance across relevant subgroups, and further establish the model’s utility in guiding individualized management decisions.

## Figures and Tables

**Table 1:** Baseline Characteristics.

Factors	Total (*n* = 244)	Stability (*n* = 83)	Instability (*n* = 161)	*p* Valve
Center, *n* (%)				
Rajavithi hospital	215 (88.1)	68 (81.9)	147 (91.3)	0.03
Tharongchang hospital	29 (11.9)	15 (18.1)	14 (8.7)	
Female, *n* (%)	184 (75.4)	67 (80.7)	117 (72.7)	0.17
Occupation, *n* (%)				
Labor	70 (28.7)	26 (31.3)	44 (27.3)	0.004
Maid	58 (23.8)	27 (32.5)	31 (19.3)	
Officer	34 (13.9)	14 (16.9)	20 (12.4)	
Other	82 (33.6)	16 (19.3)	66 (41.0)	
Dominant hand, *n* (%)				
Right	239 (98.0)	81 (97.6)	158 (98.1)	1.00
Left	5 (2.0)	2 (2.4)	3 (1.9)	
Injury hand, *n* (%)				
Right	118 (48.4)	40 (48.2)	78 (48.4)	0.97
Left	126 (51.6)	43 (51.8)	83 (51.6)	
Initial factors				
Age, year	58.5 ± 16.7	55.7 ± 14.7	59.9 ± 17.5	0.06
Age > 60 years, *n* (%)	111 (45.5)	31 (37.3)	80 (49.7)	0.07
Ulnar fracture	106 (43.4)	31 (37.3)	75 (46.6)	0.17
Dorsal angulation, degree	−10.5 ± 17.2	−6.5 ± 12.7	−12.5 ± 18.8	0.008
Dorsal angulation > 20 degree	71 (29.1)	10 (12.0)	61 (37.9)	<0.001
Intra-articular fracture, *n* (%)	98 (40.2)	18 (21.7)	80 (49.7)	<0.001
Intra-articular fracture stepping *, mm	0 [0, 2]	0 [0, 0]	0 [0, 2]	<0.001
Dorsal comminution	175 (71.7)	47 (56.6)	128 (79.5)	<0.001
Ulnar variance *, mm	3.0 [1.5, 5.0]	2.0 [1.0, 3.0]	4.0 [2.0, 5.0]	<0.001
Ulnar variance > 3 mm	101 (41.4)	14 (16.9)	87 (54.0)	<0.001
2MCP measurement ≤ 50 percentage	72 (29.5)	22 (26.5)	50 (31.1)	0.46
Radial inclination, degree	18.6 ± 7.2	22.3 ± 5.8	16.7 ± 7.1	<0.001
Radial inclination ≤ 10 degree	32 (13.1)	3 (3.6)	29 (18.0)	0.001
Radial height, mm	8.7 ± 3.3	10.3 ± 2.8	7.9 ± 3.3	<0.001
Radial height ≤ 11 mm	198 (81.1)	56 (67.5)	142 (88.2)	<0.001
Metaphyseal comminution	85 (34.8)	9 (10.8)	76 (47.2)	<0.001
Distal radioulnar joint separation	124 (50.8)	25 (30.1)	99 (61.5)	<0.001
Quality of reduction factors				
Restoration of volar cortex				
Anatomic type	92 (37.7)	48 (57.8)	44 (27.3)	<0.001
Dorsal overlapping type	93 (38.1)	27 (32.5)	66 (41.0)	
Volar overlapping type	59 (24.2)	8 (9.6)	51 (31.7)	
Volar angulation, degrees	3.2 ± 10.2	5.1 ± 6.5	2.2 ± 11.6	0.040
Volar angulation ≤ 0 degree	88 (36.1)	16 (19.3)	72 (44.7)	<0.001
Distal radioulnar joint separation	63 (25.8)	12 (14.5)	51 (31.7)	0.004
Radial translation *, mm	1 [0, 3]	0 [0, 1]	1 [0, 3]	<0.001
Three-point molding distance, mm	−1.9 ± 15.0	−2.2 ± 15.3	−1.7 ± 14.8	0.79
Factors at 1 week				
Ulnar variance *, mm	2.0 [1.0, 3.0]	1.0 [0.0, 2.0]	3.0 [1.0, 4.0]	<0.001
Ulnar variance > 3 mm	59 (24.2)	1 (1.2)	58 (36.0)	<0.001
Volar angulation, degree	1.3 ± 10.8	4.1 ± 7.0	−0.1 ± 12.1	0.004
Volar angulation ≤ 0 degree	103 (42.2)	19 (22.9)	84 (52.2)	<0.001

Data are presented as mean ± standard deviation for continuous variables and number (percent) for categorical variables. Percentages may not total 100 due to rounding. * Non-normal distributed variables are presented as median [interquartile range]. Abbreviations: 2MCP, Second metacarpal cortical percentage.

**Table 2:** Univariable Logistic Regression for Instability of Distal Radius Fractures.

Prognostic Factors	OR (95% CI)	*p* Valve
Initial factors		
Age, every 1 year	1.02 (1.00–1.03)	0.07
Ulnar fracture	1.46 (0.85–2.52)	0.17
Dorsal angulation > 20 degree	4.45 (2.14–9.27)	<0.001
Intra-articular fracture stepping, every 1 mm	1.95 (1.43–2.67)	<0.001
Dorsal comminution	2.97 (1.67–5.30)	<0.001
Ulnar variance > 3 mm	5.79 (3.02–11.13)	<0.001
2MCP measurement ≤ 50 percentage	1.25 (0.69–2.25)	0.46
Quality of reduction factors		
Restoration of volar cortex		
Anatomic reduction	Reference	-
Dorsal overlapping	2.67 (1.45–4.89)	0.002
Volar overlapping	6.95 (2.97–16.27)	<0.001
Volar angulation ≤ 0 degree	3.39 (1.81–6.35)	<0.001
DRUJ separation	2.74 (1.37–5.50)	0.004
Radial translation	1.59 (1.29–1.97)	<0.001
Three-point molding distance	1.00 (0.98–1.02)	0.79

Abbreviations: OR, odds ratio; CI, confidence interval; 2MCP, Second metacarpal cortical percentage; DRUJ, Distal radioulnar joint.

**Table 3:** Multivariable Logistic Regression and Predictive Score for Instability of Distal Radius.

Prognostic Factors	OR (95% CI)	*p* Valve	Coefficient	Score
Initial factors				
Dorsal angulation > 20 degree	3.74 (1.56–8.96)	0.003	1.32	1.5
Intra-articular fracture *, every 1 mm	2.27 (1.51–3.41)	<0.001	0.82	1
Ulnar variance > 3 mm	5.97 (2.77–12.88)	<0.001	1.79	2
Quality of reduction factors				
Restoration of volar cortex				
Anatomic reduction	Reference	-	Reference	0
Dorsal overlapping	2.34 (1.10–4.98)	0.03	0.85	1
Volar overlapping	4.35 (1.59–11.89)	0.004	1.47	2
Volar angulation ≤ 0 degree	4.78 (2.20–10.36)	<0.001	1.56	2

* The maximum value was used. A multivariable stepwise logistic regression model was developed incorporating both initial and post-reduction factors. The predictive scores were calculated by assigning weights proportional to the lowest coefficient (0.82), followed by rounding to the nearest 0.5 or integer value. The resulting pre-rounded weights were 1.61 (1.32/0.82) for dorsal angulation, 1.0 (0.82/0.82) for intra-articular fracture, 2.18 (1.79/0.82) for ulnar variance, 1.04 (0.85/0.82) for dorsal overlapping, 1.79 (1.47/0.82) for volar overlapping, and 1.90 (1.56/0.82) for volar angulation. Abbreviations: OR, odds ratio; CI, confidence interval.

**Table 4:** Clinical prediction rules based on predictive score stratification.

Instability Score	Probability Category	LR+	Sensitivity	Specificity	PPV	NPV
≤2	Low	0.19	13.7%	28.9%	27.2%	14.7%
2.5–5	Moderate	1.84	46.6%	74.7%	78.1%	41.9%
≥5.5	High	11.00	39.8%	96.4%	95.5%	45.2%

Abbreviations: LR+, positive likelihood ratio; PPV, positive predictive value; NPV, negative predictive value.

## Data Availability

The datasets used and analyzed during the current study are not publicly available due to patient confidentiality but are available from the corresponding author on reasonable request.

## Supplementary Materials

The following supporting information can be downloaded at: https://www.mdpi.com/article/10.3390/jcm14238336/s1, Table S1: Posteroanterior View Parameters; Table S2: Lateral View Parameters; Table S3: Multivariable Logistic Regression of Predictors at One Week; Figure S1: Study flow diagram; Figure S2: Discrimination plot of final model; Figure S3: Predictive Model Calibration Plot; Figure S4: The Area Under the Curve of Clinical Prediction Rule: (a) New Model, (b) Lafontaine Criteria.

## Abbreviations

The following abbreviations are used in this manuscript:

2MCP	Second Metacarpal Cortical Percentage
AUC	Area Under the Curve
CI	Confidence Interval
DA	Dorsal Angulation
DRFs	Distal Radius Fractures
DRUJ	Distal Radioulnar Joint
IAFS	Intra-articular Fracture Stepping
ICC	Intraclass Correlation Coefficient
LR+	Positive Likelihood Ratio
NPV	Negative Predictive Value
PPV	Positive Predictive Value
OR	Odds Ratio
RI	Radial Inclination
RH	Radial Height
RS	Radial Shortening
RT	Radial Translation
UF	Ulnar Fracture
UV	Ulnar Variance
