# Supplementary material for: A Prediction Model for Instability in Adult Distal Radius Fractures: Integrating Post-Reduction and Follow-Up Indicators
Source: J Clin Med. 2025 Nov 24;14(23):8336. doi: 10.3390/jcm14238336 (PMC12693056; doi:10.3390/jcm14238336)
Supplement: Supplementary file 1 [file jcm-14-08336-s001.zip › Table S1-S3.pdf]

Table S1. Posteroanterior View Parameters

Parameters	Assessment
(a) Radial Inclination (degree)	The angle formed between a line connecting the radial and ulnar margins of the distal radial articular surface and a line drawn perpendicular to the longitudinal axis of the radius.
(b) Radial Height (mm)	The vertical distance from the most distal point of the radial articular surface to a line perpendicular to the radial axis through the ulnar margin of the distal articular surface.
(c) Ulnar Variance (mm)	The difference between the distal ulnar articular surface and the ulnar margin of the distal radial articular surface, measured along a line perpendicular to the longitudinal axis of the radius.
(d) Ulnar Fracture	The presence of a fracture fragment involving the distal ulna.
(e) Metaphyseal Comminution	The presence of more than two fracture fragments within the metaphyseal region of the distal radius.
(f) DRUJ Separation	Sigmoid notch–ulnar head misalignment was observed.
(g) Radial Translation (mm)	The horizontal displacement between the radial margin of the distal fracture fragment and that of the proximal fragment.
(h) Second Metacarpal Cortical Percentage (2MCP)	The percentage was calculated as the difference between the outer cortical diameter and the intramedullary diameter at the narrowest point of the second metacarpal mid-diaphysis, normalized to the outer cortical diameter.
(i) Radial Shortening (mm)	The vertical distance from the most distal point of the radial articular surface to the ulnar margin of the distal radius, measured parallel to the longitudinal axis of the radius.

Table S2. Lateral View Parameters

Parameters	Assessment
(a) Volar/ Dorsal Angulation (degree)	The angle between a line connecting the dorsal and volar edges of the distal radial articular surface and a line perpendicular to the longitudinal axis of the radius. Dorsal angulation is recorded as a negative value of volar angulation.
(b) Articular Step-Off (mm)	The vertical displacement between the most distal points of the volar and dorsal articular surfaces of the distal radius, assessed relative to the longitudinal axis of the radius.
(c) Dorsal Comminution	The presence of free-floating fracture fragments along the dorsal cortex of the distal radius.
(d) Restoration of Volar Cortex	*Anatomic type*: the volar cortex of the distal fragment aligns with that of the proximal fragment.<br>*Dorsal overlapping type*: the volar cortex of the distal fragment lies dorsal to the proximal cortex.<br>*Volar overlapping type*: the volar cortex of the distal fragment lies volar to the proximal cortex.
(e) Three-Point Molding Distance (mm)	The distance between a line at the fracture site on the volar cortex and a line at the apex (or narrowest point) of the volar splint. The measurement is positive when the splint's narrowest point is proximal to the fracture site.

Table S3 Multivariable Logistic Regression of Predictors at One Week

Prognostic factors	Multivariable	
	**OR (95 CI)**	***P* valve**
Ulnar variance > 3 mm	9.67 (1.22-76.63)	0.03
Volar angulation ≤ 0 degree	1.84 (0.66-5.14)	0.24

Abbreviations: OR, odds ratio; CI, confidence interval
