# Supplementary figures and images for: A Prediction Model for Instability in Adult Distal Radius Fractures: Integrating Post-Reduction and Follow-Up Indicators
Source: J Clin Med. 2025 Nov 24;14(23):8336. doi: 10.3390/jcm14238336 (PMC12693056; doi:10.3390/jcm14238336)

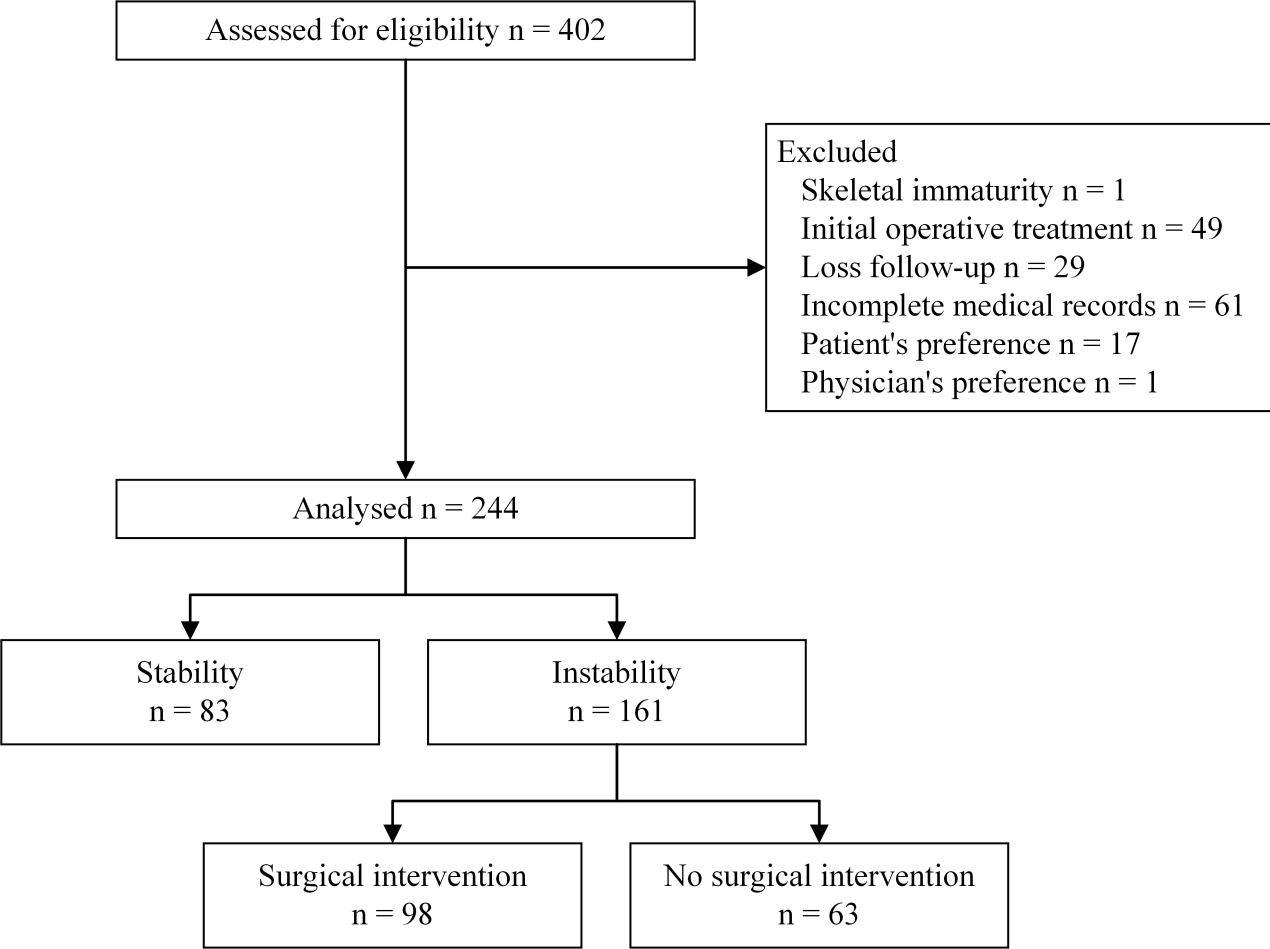

Supplement: Supplementary file 1 [file jcm-14-08336-s001.zip › Figure S1.pdf]

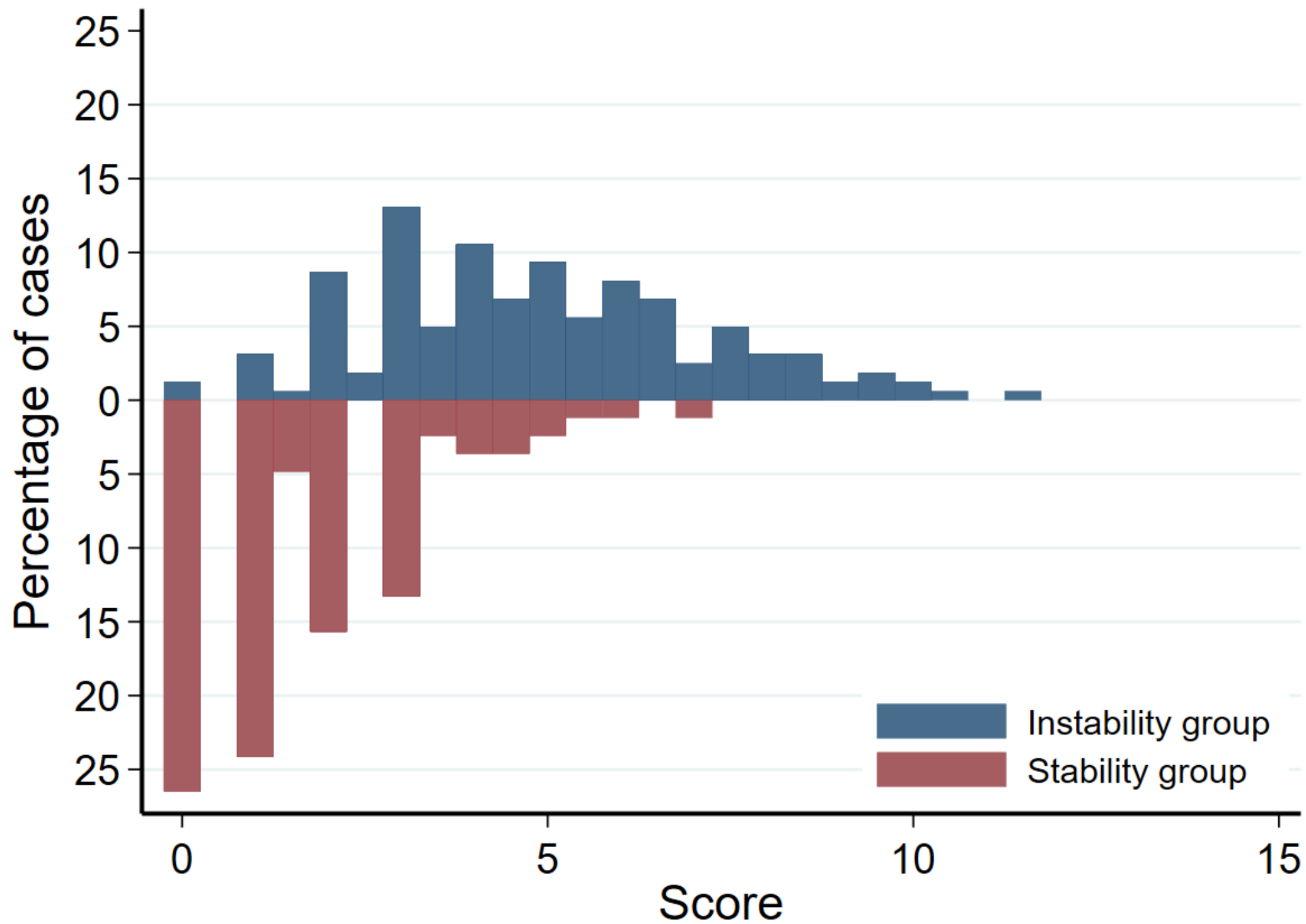

Supplement: Supplementary file 1 [file jcm-14-08336-s001.zip › Figure S2.pdf]

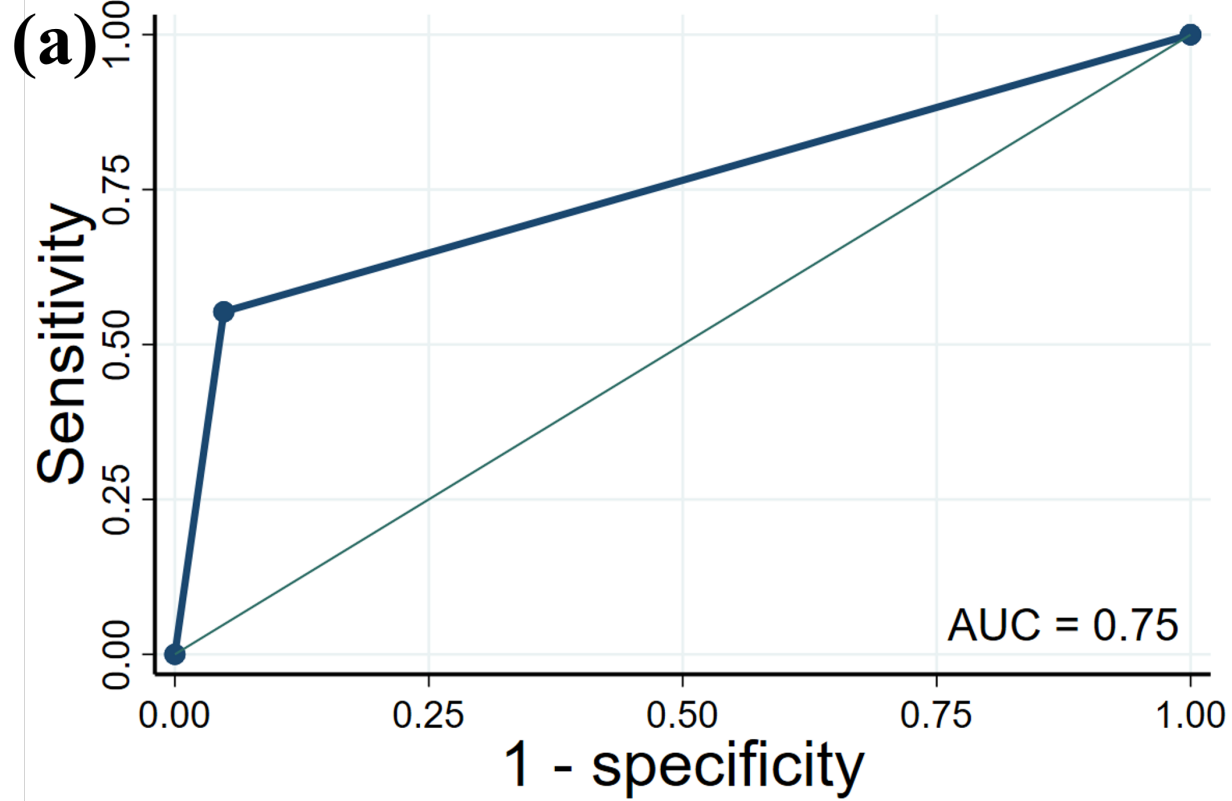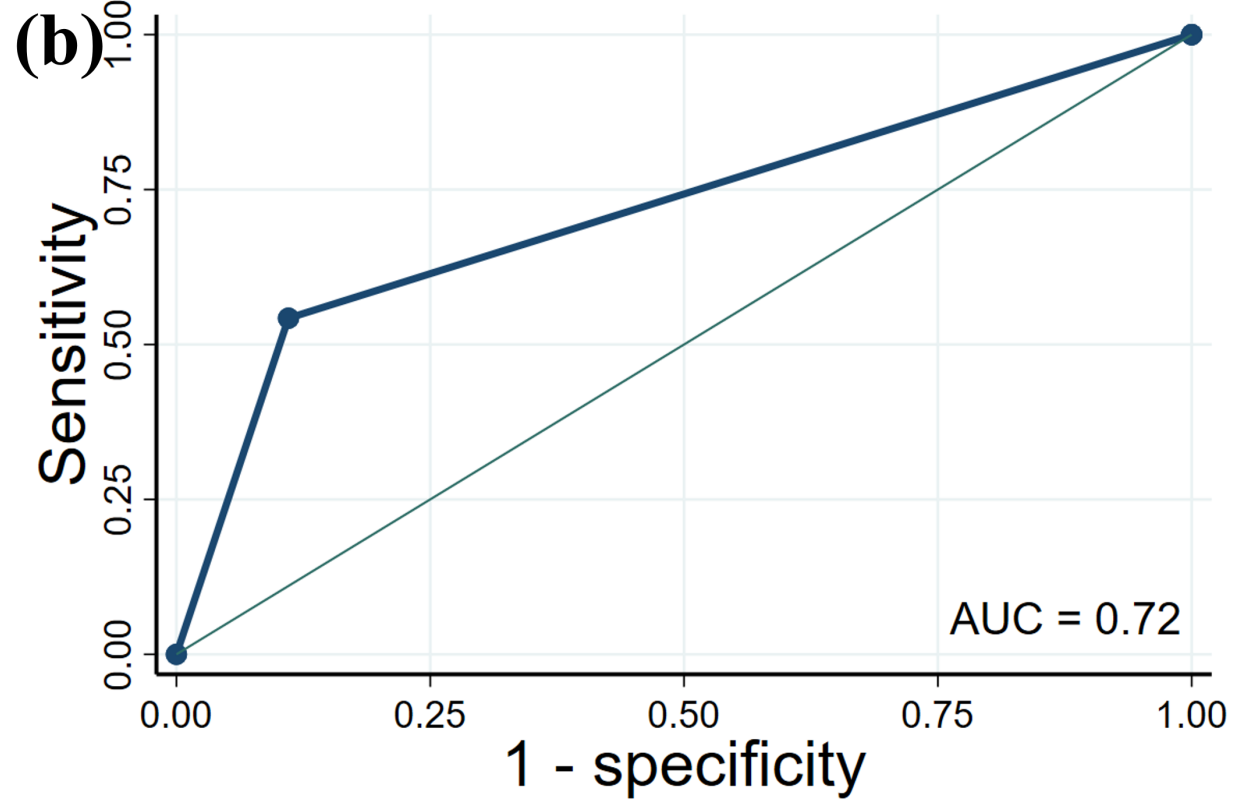

Supplement: Supplementary file 1 [file jcm-14-08336-s001.zip › Figure S4.pdf]
